# Peer Review of “The Role of Animal-Assisted Therapy in Enhancing Patients’ Well-Being: Systematic Study of the Qualitative and Quantitative Evidence”

**DOI:** 10.2196/56440

**Published:** 2024-03-18

**Authors:** Anonymous

**Keywords:** animal-assisted therapy, pet therapy, outcome assessment, policies, systematic study

*This is a peer-review report submitted for the paper “The Role of Animal-Assisted Therapy in Enhancing Patients’ Well-Being: Systematic Study of the Qualitative and Quantitative Evidence.”*

## Round 1 Review

Dear Authors,

First of all, your work’s [1] topic is up-to-date and meticulously prepared. However, I still have a few questions/suggestions:

1. In the Identification section, the total number of articles obtained from each database is given. It is recommended to give separate numbers for each.

2. In the box below, the numbers are given as a total, but it may be more appropriate to give separate data for each item.

3. Can keywords be schematized in accordance with PICOS (Population, Intervention, Comparison, Outcomes, Study Design) in the literature review section?

Table …: Keywords used while browsing.

Population

Intervention

Comparison

Outcomes

Study design

4. Has the quality of evidence been evaluated? If so, how was it done? This process can be explained by creating such a subtitle.

- How did you reduce the risk of bias in studies? Were the articles evaluated and scored separately among authors? Have these scores been analyzed?
- By whom and how was the screening done? I think that the most important limitation of this study is that it was scanned by a single person.

5. In the section where general information is given for the last 16 articles, can it be added which disciplines are studied in particular? Since this subject is studied by various job groups, adding this information can enrich the data. If the mentioned situations are arranged, your article will contribute more to the literature.

6. What has been studied in previous systematic reviews? What are the original aspects of this work?

I include below some systematic review studies that may be relevant to the subject:

- Stern C, Lizarondo L, Carrier J, et al. The experiences and effectiveness of canine-assisted interventions (CAIs) on the health and well-being of older people residing in long-term care: a mixed methods systematic review protocol. PROSPERO. 2020. URL: https://www.crd.york.ac.uk/prospero/display_record.php?ID=CRD42020161235
- Whear R, McGill P, Orr N, et al. What are the effects of ‘robopets’ on the health and wellbeing of older people resident in care homes? A systematic review of qualitative and quantitative evidence. PROSPERO. 2017. URL: https://www.crd.york.ac.uk/prospero/display_record.php?ID=CRD42017081794
- Nabi N, McAloney-Kocaman K, Fleming M, Bain S. A systematic review exploring the effectiveness of animal-assisted therapy in improving the psychological well-being of incarcerated individuals. PROSPERO. 2022. URL: https://www.crd.york.ac.uk/prospero/display_record.php?ID=CRD42022314341

If the mentioned situations are arranged, your article will contribute more to the literature.

I wish you good luck in your work.

## Abbreviations

PICOS: Population, Intervention, Comparison, Outcomes, Study Design
